# Recurrent severe viral infection in a child with inherited complete TBK1 deficiency

**DOI:** 10.70962/jhi.20250058

**Published:** 2025-12-03

**Authors:** Sara Sebnem Kilic, Shuxiang Zhao, Zhiyong Liu, Yasin Karali, Koray Yalcin, Aaron Bodansky, Debanjana Chakravarty, Michael Wilson, Jean-Laurent Casanova, Shen-Ying Zhang

**Affiliations:** 1 Department of Pediatric Immunology-Rheumatology, Bursa Uludag University, Medical Faculty, Bursa, Turkey; 2 St. Giles Laboratory of Human Genetics of Infectious Diseases, Rockefeller Branch, Rockefeller University, New York, NY, USA; 3 Department of Pediatric Hematopoietic Stem Cell Transplantation Unit, Bahcesehir University, Medical Faculty, Istanbul, Turkey; 4 Division of Critical Care, Department of Pediatrics, University of California, San Francisco, San Francisco, CA, USA; 5 Weill Institute for Neurosciences, University of California, San Francisco, San Francisco, CA, USA; 6 Department of Neurology, University of California, San Francisco, San Francisco, CA, USA; 7 Laboratory of Human Genetics of Infectious Diseases, Necker Branch, Institut National de la Santé et de la Recherche Médicale (INSERM) U1163, Necker Hospital for Sick Children, Paris, France; 8 Paris Cité University, Imagine Institute, Paris, France; 9 Howard Hughes Medical Institute, New York, NY, USA; 10 Department of Pediatrics, Necker Hospital for Sick Children, APHP, Paris, France

## Abstract

TANK-binding kinase 1 (TBK1) acts at the crossroads of various host immune pathways. Autosomal dominant (AD) and recessive (AR) TBK1 deficiencies have been reported in human patients with herpes simplex encephalitis and SARS-CoV-2 pneumonia (AD deficiency) or systemic inflammation (AR deficiency). We describe here a Turkish boy, born to consanguineous parents and homozygous for a loss-of-function mutation of *TBK1*, who had experienced recurrent vesicular skin eruptions presumably triggered by herpesviral infection and five episodes of viral pneumonia since the age of 2 mo. Whole-exome sequencing identified a biallelic mutation of the *TBK1* gene (*NM_013254.4*: c.922C>T, p.Arg308Ter). The induction of *IFNB*, *IFNL1*, *IFIT1*, and *IL6* was abolished or severely impaired in SV40-fibroblasts from the patient following stimulation of the TLR3 or RIG-I/MDA5-RIG-I pathways. The patient underwent stem-cell transplantation, but unfortunately succumbed to suspected post-viral acute disseminated encephalomyelitis at the age of 2.5 years. AD or AR TBK1 deficiency should be considered in patients with severe viral infections.

## Teaser

Kilic et al. reported a child with a homozygous TBK1 mutation previously linked to autoinflammation, suggesting its role in recurrent severe viral infections; the patient died from acute disseminated encephalomyelitis.

## Introduction

TANK-binding kinase 1 (TBK1) is a ubiquitously expressed serine/threonine kinase. It acts at the crossroads of various signaling pathways, including type I and type III interferon (I-IFN, III-IFN) induction, autophagy, and receptor-interacting serine/threonine-protein kinase 1 (RIPK1)–mediated cell death (1). TBK1 was originally shown to be an important signaling component for the induction of I-IFNs and inflammatory cytokines downstream from various sensors, including TLR3-TRIF, RIG-I/MDA5-MAVS, and cGAS-STING (2, 3). TBK1 has also been shown to inhibit RIPK1-mediated necroptotic and apoptotic cell death, and to regulate autophagy (4, 5, 6, 7). Biochemical studies *in vitro* and mouse studies *in vivo* have placed TBK1 at an intriguing key position at the intersection of antiviral type I immunity and inflammation. Complete deficiencies of TBK1 are embryo-lethal in mice. The role of human TBK1 *in vivo* in natural conditions of infection has begun to be clarified, following the discovery of human inborn errors of TBK1 (8, 9, 10, 11, 12, 13).

Human autosomal dominant (AD) TBK1 deficiency was first reported in 2012, in two children with herpes simplex encephalitis (HSE) (8). These children were heterozygous for missense loss-of-function (LOF) mutations. Dominance operated by negative dominance in one child and haploinsufficiency in the other. AD TBK1 deficiency impairs TLR3-dependent I-IFN production in fibroblasts, which can be used as a surrogate for brain-resident cells. This deficiency increases the susceptibility of fibroblasts to HSV-1, providing a plausible mechanism for HSE (10). Impaired TBK1-mediated autophagy during HSV-1 infection may also contribute to HSE pathogenesis (11). Heterozygosity for predictedLOF (pLOF) mutations of *TBK1* was subsequently shown to be linked to neurodegenerative diseases, such as amyotrophic lateral sclerosis (ALS) and frontotemporal dementia (FTD) in particular (12). It has been suggested that TBK1 haploinsufficiency would result in impaired autophagy, contributing to the pathogenesis of ALS and FTD, but this hypothesis has been only partially tested. More recently, AD TBK1 deficiency was reported to underlie COVID-pneumonia in some other patients (13).

Autosomal recessive (AR) TBK1 deficiency was first reported in 2021, in four patients homozygous for LOF mutations of *TBK1* presenting systemic autoinflammation with no prior history of severe viral infection (14). Studies on fibroblasts from these patients showed that AR TBK1 deficiency led to a failure to inhibit TNFR-RIPK1–mediated necroptosis and apoptosis, providing a plausible mechanism for autoinflammation in these patients. Nevertheless, an abolition of I-IFN induction following TLR3 stimulation was observed in the patients’ fibroblasts, and responses to RIG-I/MDA5-MAVS stimulation were impaired, but not abolished. It was suggested that residual RIG-I/MDA5-MAVS pathway-mediated I-IFN production might compensate for the lack of TLR3-IFN response during peripheral viral infections, accounting for the absence of a prior history of severe viral infections in these four patients (14, 15). However, one child homozygous for pLOF mutations of *TBK1* died from critical COVID-19 (16). We describe here a case of AR TBK1 deficiency in a child with recurrent vesicular skin eruptions likely triggered by herpes virus infection, recurrent viral pneumonia, who ultimately died from encephalomyelitis with a presumably viral origin.

## Results

### Clinical case report

**Figure 1.**
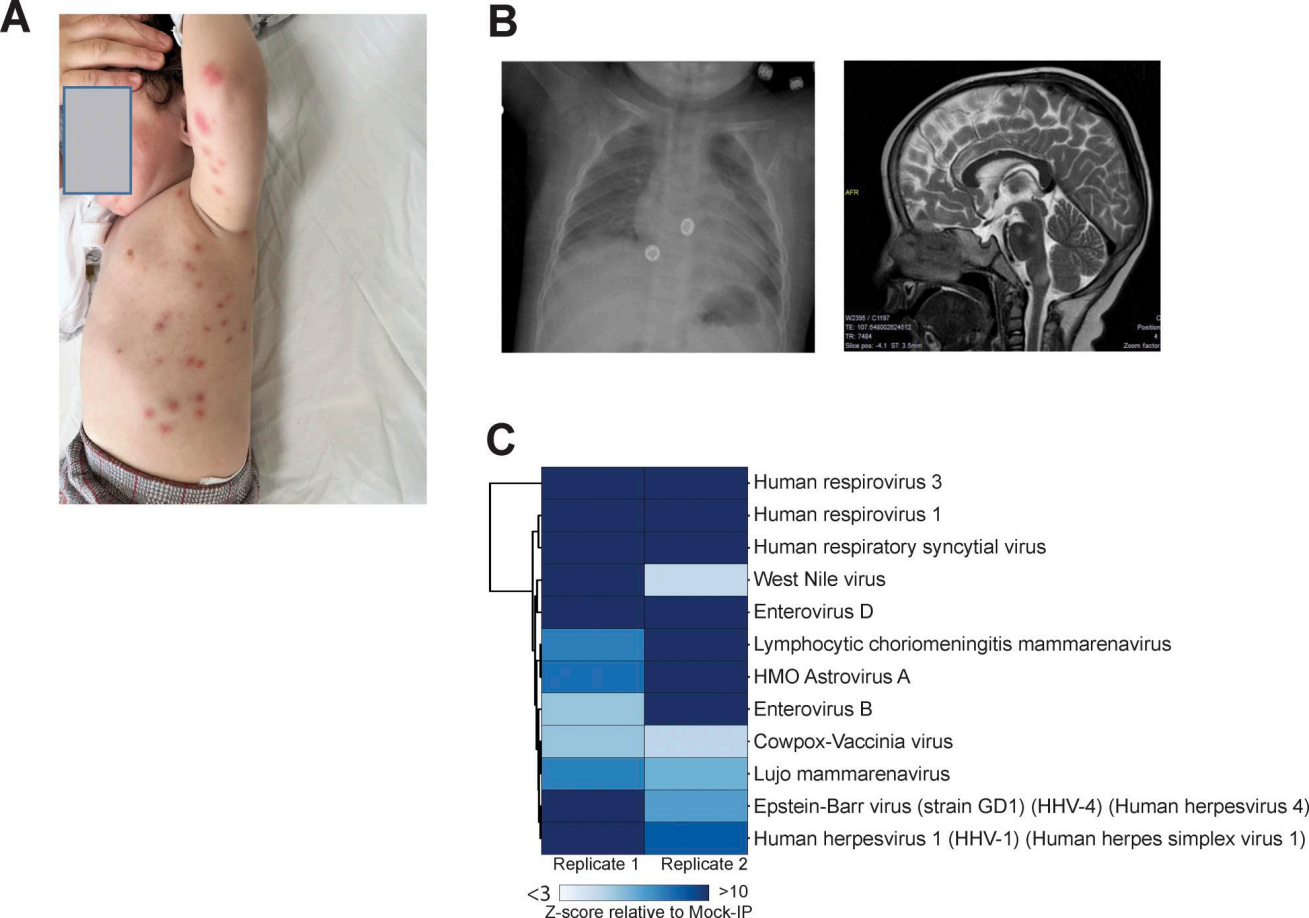
**Skin, lung, and brain lesions of the patient. (A)** Skin lesions observed during one episode of herpes virus skin eruptions in the patient. **(B)** Chest x ray taken during an episode of adenovirus pneumonia (left panel), and brain MRI revealed lesions involving the cerebral cortex and pons, suggestive of encephalomyelitis (axial and sagittal views, right panel).” Brain MRI showing brain lesions involving the cerebral cortex and pons suggestive of encephalomyelitis (right panel). **(C)** VirScan test for antibodies against a wide range of viruses in the plasma of the patient. Hierarchically clustered (Pearson) heatmap showing PhIP-Seq antibody enrichment (z-score relative to mock immunoprecipitation [IP]) for each of the 12 viruses detected in the patient.

We studied a Turkish boy born to first-degree cousins. The patient was first seen at 6 mo of age, when he presented with recurrent erythematous rashes, initially appearing in the genital area at 2 mo of age and rapidly spreading to the entire body. Intermittent vomiting and diarrhea began at the age of 3 mo. There was no family history of similar manifestations and the patient had a healthy older brother. On physical examination, the patient was found to have red, elevated skin eruptions with vesicles (Fig. 1 A) and a fever. No hepatomegaly, splenomegaly, or neurological abnormalities were observed. Allergy tests yielded normal results. Blood cultures revealed the presence of *Staphylococcus hominis*, leading to treatment with cefotaxime. The patient was hospitalized 14 times over the following 2 years for persistent vomiting, diarrhea, red and elevated skin eruptions, fever, high levels of acute-phase reactants, and leukocytosis. Notably, leukocytosis was observed even when the patient appeared otherwise healthy.

The pathological evaluation of a skin biopsy specimen revealed leukocytoclastic, diffuse inflammation with rare eosinophils. Attempts to isolate the virus and direct testing for antigen detection yielded negative results, and tests for the presence of HSV DNA in blood, based on the molecular diagnostic techniques available at the hospital, were also negative. Acyclovir treatment was nevertheless initiated, based on a presumed diagnosis of herpes virus infection. This treatment led to a complete resolution of skin rashes within 1 wk. A similar pattern of rapid resolution was consistently observed with each subsequent course of acyclovir treatment. On one occasion, the mother refused to allow her child to be treated with acyclovir, resulting in the persistence of skin lesions. These lesions ultimately resolved within 2 days of acyclovir treatment being reinitiated.

The patient was also hospitalized five times for viral pneumonia, following infections with both adenovirus and rhinovirus (at 8 and 12 mo), or with respiratory syncytial virus (RSV) (at 10, 11, 18 mo). During each episode of viral pneumonia, a swab sample taken from the respiratory tract was analyzed by PCR to identify the causal virus. For each episode of pneumonia, the diagnosis was confirmed by chest x ray (Fig. 1 B). The patient received symptomatic treatment during each episode of viral pneumonia and responded well to treatment. He did not experience SARS-CoV-2 infection and remained seronegative for antibodies against SARS-CoV-2 until the age of 2.5 years.

Laboratory tests performed at 6 mo of age revealed leukocytosis, anemia, normal serum immunoglobulin levels, and low CD8^+^ T cell counts (Table 1). Before the initiation of intravenous immunoglobulin (IVIG) treatment at 6 mo of age, serological tests revealed the presence of IgG antibodies against HSV-1, varicella zoster virus, and cytomegalovirus (CMV). VirScan on plasma from a blood sample taken at the age of 2 years revealed that the patient was positive for antibodies against HSV-1, RSV, human respiroviruses 1 and 3, and several other viruses (Fig. 1 C), suggesting past infections consistent with the recurrent episodes of viral infection in the patient. However, caution was required in the interpretation of the VirScan data due to the prior treatment of the patient with IVIG, and the presence of antiviral antibodies can also be due to the past vaccination history (e.g., varicella zoster virus).

**Table 1.** Clinical laboratory findings for the patient with TBK1 deficiency

Laboratory parameters	Result (normal range)	After HSCT
**Hemogram**		
Leukocytes (cells/μl)	18,000 (5,430–13,470)	5,270
Neutrophils (cells/μl)	6,600 (1,470–6,280)	3,540
Lymphocytes (cells/μl)	9,050 (1,860–7,320)	1,160
Monocytes (cells/μl)	1,190 (310–1020)	510
Eosinophils (cells/μl)	996 (50–740)	60
Basophils (cells/μl)	58 (10–50)	0
Hemoglobin (g/L)	9.2 (11.6–14.6)	12.4
Platelets (cells/μl)	691,000 (206,400–434,300)	145,000
**Immunoglobulin levels**		
IgG (mg/dl)	752 (294–1165)	708
IgM (mg/dl)	231 (33–154)	155
IgA (mg/dl)	57 (13.5–72)	61
Total IgE (IU/ml)	7	9
**Lymphocyte immunophenotype**		
CD3^+^ %	52.5 (52–77)	51.2
CD3^+^ (cells/μl)	4,751 (1,850–5,960)	594
CD4^+^ %	41.3 (30–58)	33
CD4^+^ (cells/μl)	3,730 (1,140–3,800)	383
CD8^+^ %	8.9 (12–27)	8.1
CD8^+^ (cells/μl)	805 (540–1970)	94
CD4^+^/CD8^+^	4.6 (1.9–2.1)	4.07
HLA DR	35.5 (4–27)	32
CD19^+^ %	34.1 (15–28)	21.2
CD19^+^ (cells/µl)	3,086 (640–1,960)	246
CD3^−^CD16^+^CD56^+^ %	13.1 (3–24)	9.9
CD3^−^CD16^+^CD56^+^ (cells/μl)	1,185 (150–1,330)	115
CD19^+^IgD^−^CD27^+^ %	0.2 (1.1–20.3)	0.9
CD19^+^IgD^−^CD27^+^ (cells/µl)	6 (6.2–81.2)	1
CD19^+^IgD^+^CD27^−^ %	31.2 (57.4–92.1)	28.9
CD19^+^IgD^+^CD27^−^ (cells/µl)	962 (139.2–1,127.1)	33
**CD3** ^**+**^ **CD4** ^**+**^ ** (41.2%) gate**		
CD45RA %/(MFI)	91.1 (64–93)/(1,927.97)	-
CD45RO %/(MFI)	7.5 (5–18)/(7,246.01)	-
**Antibody level**		
Anti-HbS (IU/ml)	>1,000	-
**Acute-phase reactants**		
CRP (mg/dl)	27.7 (0–0.5)	0.27
Sedimentation (mm/h)	17	14
Serum amyloid A concentration (mg/L)	14.6 (0–6.8)	0.5
**Other**		
NBT %	96	-
EBV VCA IGM (U/ml)	Negative	-
EBV VCA IGG (U/ml)	60.1 (positive) (cutoff: 18)	-
EBV EBNA IGG (U/ml)	20.4 (positive) (cutoff: 18)	-
EBV PCR	Negative	-
CMV IGM (U/ml)	Negative	-
CMV IGM (U/ml)	31 (positive) (cutoff: 5.99)	​
CMV-DNA PCR	Negative	-
HSV-1 IgG (serum) (U/ml)	69 (positive) (cutoff: 1.10)	-
HSV-2 (serum) (U/ml)	Negative	-
Varicella IgM (U/mM)	Negative	-
Varicella IgG (U/ml)	1,249 (positive) (cutoff: 165)	-
HIV Ag/Ab	Negative	-
Anti-HCV	Negative	-
ANA (antinuclear antibodies)	Negative	-
ANA panel	Negative	-
Anti LKM-1	Negative	-
Anti-dsDNA	Negative	-
AMA-M2	Negative	-
ASMA	Negative	-
ANCA panel	Negative	-
p-ANCA	Negative	-
Anti-tissue transglutaminase IgA (U/ml)	0.8 (<10 normal)	​
Anti-tissue transglutaminase IgG (U/ml)	1.9 (<10 normal)	-
Sweat chloride test (mmol/L)	22.3 (<40 normal)	-

HSCT, hematopoietic stem-cell transplantation; NBT, Nitroblue tetrazolium; dsDNA, double-stranded DNA; ASMA, anti-smooth muscle antibody; ANCA, antineutrophil cytoplasmic antibodies; VCA, Viral capsid antigen; HCV, Hepatitis C virus; EBNA, EBV nuclear antigen.

**Figure 2.**
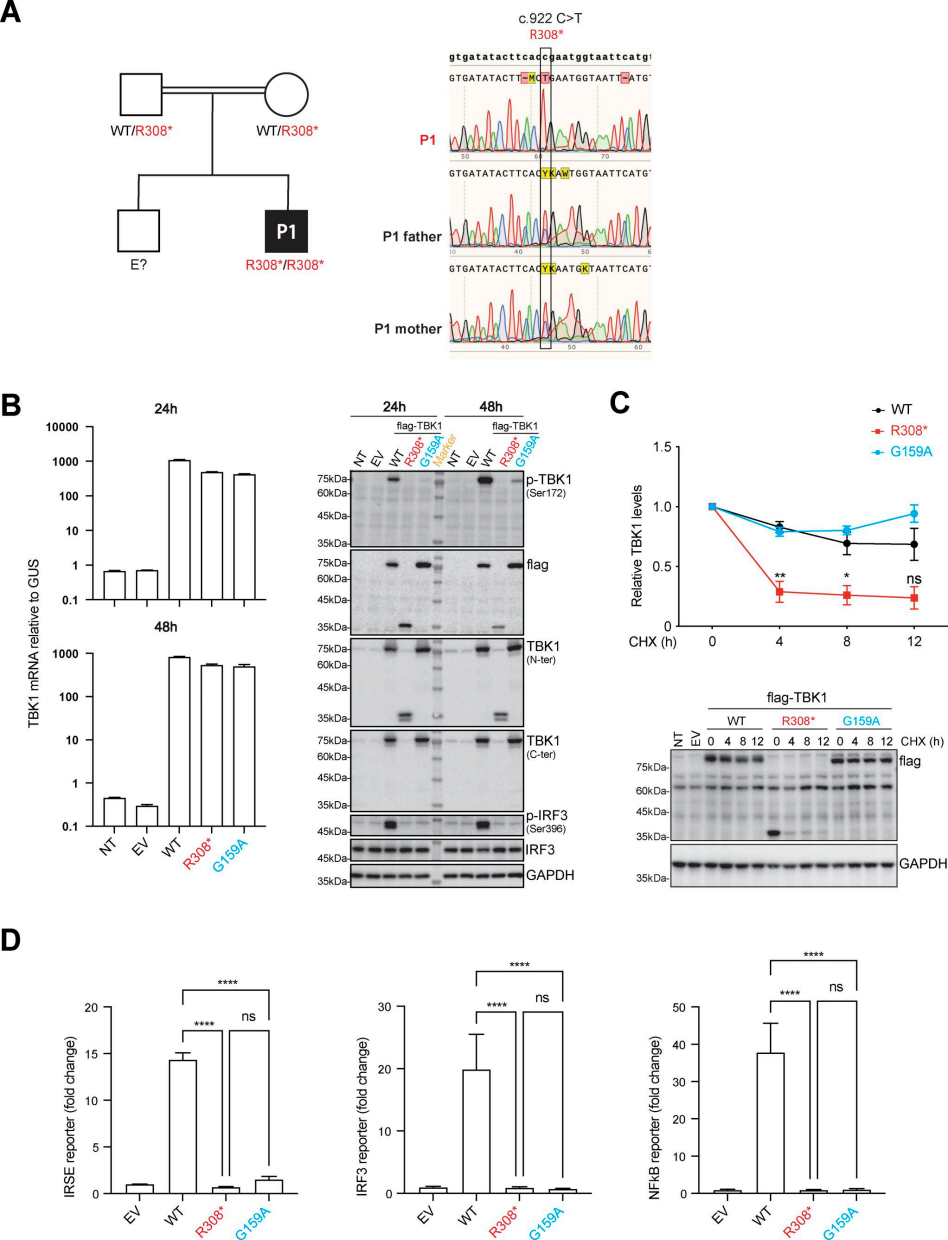
**Homozygosity for a LOF *TBK1* mutation in the patient. (A)** Family pedigree showing segregation of the *TBK1* mutation. PCR products were amplified from genomic DNA extracted from the granulocytes of P1 and both parents and subjected to Sanger sequencing. **(B)***TBK1* mRNA levels (upper panel) were determined by RT-qPCR on HEK293T cells 24 and 48 h after transfection with empty vector (EV), WT, and mutant TBK1 constructs. Western blot analysis was performed to assess the levels of protein for TBK1 (lower panel), autophosphorylated TBK1 (p-TBK1, Ser172), IRF3, and autophosphorylated IRF3 (p-IRF3, Ser396) in HEK293T cells at 24 and 48 h after transfection with EV, N-terminally flag-tagged WT, and mutant TBK1 constructs. The results shown are representative of three independent experiments. **(C)** Pulse-chase analysis of WT and mutant TBK1 protein stability. HEK293T cells were transfected with flag-tagged WT or mutant TBK1 expression plasmids for 24 h. Cells were then treated with cycloheximide (CHX; 100 ng/ml) for the indicated time points to inhibit protein synthesis, followed by western blot analysis (bottom). TBK1 protein levels were quantified by densitometry, normalized to GAPDH, and plotted over time (top). Data shown are representative of three independent experiments. The data shown are the means ± SEM of three independent experiments. P values were obtained by one-way ANOVA with Tukey’s multiple comparisons tests, and the P values for the 4-, 8-, and 12-h time points are indicated for the comparison of P1’s cells with control cells. ns; P > 0.05; *P < 0.05; and **P < 0.01. **(D)** ISRE, IRF3, and NF-κB promoter-driven luciferase reporter assays were performed on HEK293T cells 24 h after transfection with ISRE, IRF3, or NF-κB reporter plasmids along with EV, WT, and mutant TBK1 constructs. Luciferase activity was measured to assess TBK1-mediated activation. The results shown are representative of three independent experiments. The data shown are the means ± SEM of three experiments with three biological replicates, P values were obtained by one-way ANOVA with Tukey’s multiple comparisons tests. ns; P > 0.05, ****P < 0.0001. Source data are available for this figure: SourceData F2.

Genetic testing by whole-exome sequencing revealed homozygosity for a mutation of the *TBK1* gene (NM_013254.4):c.922C>T (p.Arg308Ter), which was confirmed by Sanger sequencing (Fig. 2 A). IVIG was administered every 3 wk, and acyclovir prophylaxis was used to manage the skin lesions. These treatments reduced the frequency of hospitalization, but hematopoietic stem-cell transplantation was nevertheless performed at the age of 2.5 years, due to recurrent severe viral pneumonia attacks and a suspicion that the abnormal immune cell profile resulting from TBK1 deficiency might have contributed to these infections. The patient received preparative treatment with a regimen including treosulfan, fludarabine, cyclophosphamide, and antithymocyte globulin. He then received an infusion of 7.5 × 10^6^/kg CD34-positive peripheral stem cells in a total volume of 65 ml from a 9/10 matched unrelated donor. Methotrexate and cyclosporine were used for graft-versus-host disease (GVHD) prophylaxis. Chimerism rates reached 100% in the third week after stem-cell transplantation. Neutrophil engraftment occurred on day 13 after transplantation, and platelet engraftment occurred on day 14. On day 15 after transplantation, GVHD was suspected due to fever and a widespread maculopapular rash. Following immunosuppressive treatments, including steroids, cyclosporine, and mycophenolate mofetil, the symptoms of GVHD regressed significantly, and the patient was discharged on day 38 after transplantation.

However, on day 50 after stem-cell transplantation, the patient was readmitted to the transplantation center due to vomiting, left-sided weakness, and left facial paralysis. The patient had a body temperature of 37°C. Multifocal involvement observed in the patient’s cranial MRI, with particularly intense involvement in the brainstem causing mass effect and lacking contrast enhancement, raised the suspicion of post-viral acute disseminated encephalomyelitis (ADEM) (Fig. 1 B). The patient’s sudden onset of neurological symptoms followed by rapid death was presumed to be due to compression of the brainstem respiratory center. Laboratory tests revealed that the complete blood cell count and acute-phase reactant levels were within normal limits. The patient’s neurological condition progressively deteriorated, with the disappearance of swallowing reflexes and worsening respiratory function. Unfortunately, due to the possibility of high intracranial pressure, it was not possible to perform a lumbar puncture for cerebrospinal fluid (CSF) analysis. The patient died on the first day in intensive care, 52 days after transplantation, following sudden cardiac and respiratory arrest. Despite achieving complete chimerism after transplantation, the patient succumbed to presumed post-viral ADEM or ADEM from unknown cause.

### Molecular and cellular characterization of the *TBK1* mutation

Given the history of recurrent severe diseases with confirmed or suspected viral triggers in this patient, we focused on characterizing the antiviral I-IFN–related function of the patient’s TBK1 variant relative to that of wild-type (WT) TBK1. We investigated the expression and activity of the R308* mutant TBK1 in transiently transfected HEK293T cells. Similar levels of *TBK1* mRNA were detected in cells transfected with plasmids encoding Flag-tagged mutant TBK1 and in cells transfected with plasmids encoding the WT TBK1 (Fig. 2 B). However, the R308* TBK1 was produced as a C-terminally truncated protein that could be detected with an antibody specific for the N-terminus of TBK1 or for the Flag tag, but not with an antibody specific for the C-terminus of TBK1. Moreover, the levels of R308* decreased rapidly between 24 and 48 h after transfection, suggesting that it might be inefficiently translated or undergo enhanced post-translational degradation. We assessed the stability of mutant and WT TBK1 proteins in transiently transfected HEK293T cells following treatment with the protein synthesis inhibitor cycloheximide. The R308* protein exhibited a significantly shorter half-life compared to both G149A and WT TBK1, indicating a rapid degradation of the R308* mutant TBK1 protein (Fig. 2 C).

Consistent with the abnormal expression of the R308* mutant protein, no autophosphorylation of TBK1 or phosphorylation of IRF3 was detected in this overexpression system, contrasting with the results obtained for WT TBK1 (Fig. 2 B). Finally, the WT TBK1 induced high levels of ISRE-, IRF3-, and NF-κB–dependent luciferase induction, whereas no such activity was observed in HEK293T cells transiently transfected with R308* TBK1 (Fig. 2 D). G159A TBK1, a previously reported LOF TBK1 variant found in a patient with HSE, was included as a negative control for TBK1 activity in this experiment; it gave normal levels of a LOF in all assays performed. Thus, R308* TBK1 is produced as a C-terminally truncated protein that is prone to degradation and displays a LOF for TBK1 autophosphorylation, IRF3 phosphorylation, and ISRE/IRF3/NF-κB activation following plasmid transfection-mediated transient overexpression.

**Figure 3.**
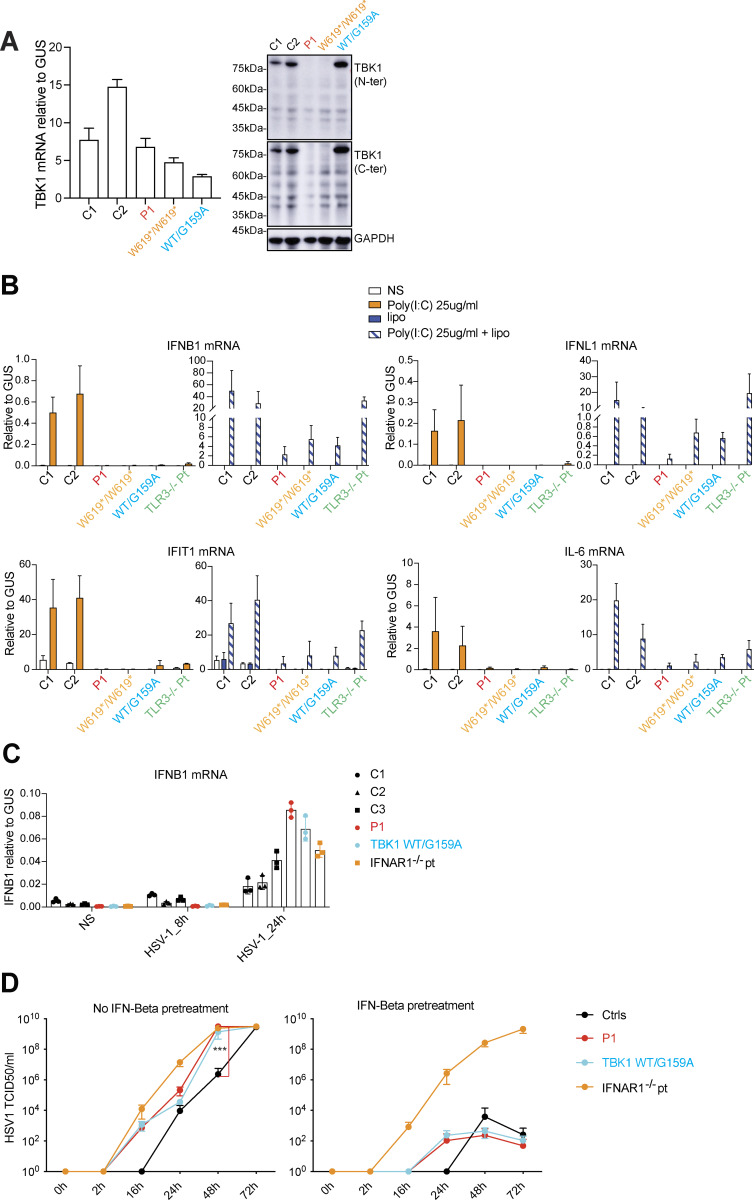
**Impaired induction of IFNs via TBK1-mediated pathways in the patient’s SV40-fibroblasts. (A)** TBK1 mRNA levels (left panel) were measured by RT-qPCR in fibroblasts (SV40-fibroblasts) from healthy controls (C1, C2), P1, and two other TBK1-deficient patients (WT/G159A, W619*/W619*), and an immunoblot analysis of endogenous TBK1 protein levels (right panel) was performed with antibodies against the N-terminus and C-terminus of TBK1. The results shown are representative of three independent experiments. **(B)** SV40-fibroblasts from healthy controls (C1, C2), P1, two other TBK1 patients (WT/G159A, W619*/W619*), and a TLR3^−/−^ HSE patient were left unstimulated (NS) or were stimulated with poly (I:C) alone, Lipofectamine alone (Lipo), or both (poly (I:C)+Lipo), for 6 h. The relative expression levels of *IFNB1*, *IFNL1*, *IFIT1*, and *IL6* were measured by RT-qPCR. The results shown are from three independent experiments. **(C)***IFNB* mRNA levels were measured by RT-qPCR in SV40-F from healthy controls (*n* = 3), P1, and HSE patients with TBK1-deficient (WT/G159A) and AR IFNAR1 deficiencies. Cells were either left uninfected (NS) or infected with HSV-1 (KOS strain, MOI = 1) for 24 h. Data represent the means of three independent experiments. **(D)** SV40-F from healthy controls (Ctrls, *n* = 3), P1, and HSE patients with TBK1-deficient (WT/G159A) and AR IFNAR1 deficiencies were either left untreated or pretreated with IFN-β for 24 h, followed by infection with HSV-1 (MOI = 0.001). Viral replication was assessed at the indicated time points post-infection using the TCID_50_ virus titration method. Data represent means ± SEM from three independent experiments. Statistical analysis was performed using one-way ANOVA followed by Tukey’s multiple comparisons test; P values at 48 h after infection compare P1’s cells to controls. ***P < 0.0001. Source data are available for this figure: SourceData F3.

We then investigated TBK1 levels and the production of mRNAs encoding IFNs (*IFNB*, *IFNL1*), one interferon-stimulated gene (ISG) (*IFIT1*), and another cytokine (*IL*6) in SV40-transformed dermal fibroblasts (SV40-fibroblasts), as a surrogate for tissue cells, following stimulation of the TLR3 or RIG-I/MDA5-MAVS pathways (17). TBK1 mRNA levels were normal in SV40-fibroblasts from the patient (P1), but no TBK1 protein was detected in these cells (Fig. 3 A). As in cells from a previously reported patient with recessive complete TBK1 deficiency (W619*/W619*) (14), a patient with HSE and a dominant TBK1 deficiency (G159A/WT) (8), and a patient with recessive complete TLR3 deficiency (18), but contrasting with cells from two healthy controls, the production of *IFNB*, *IFNL1*, *IFIT1* and *IL6* was abolished in SV40-fibroblasts from P1 following the addition of extracellular poly (I:C), which stimulates the TLR3 pathway (Fig. 3 B).

Interestingly, following intracellular transfection with poly (I:C), which stimulates the RIG-I/MDA5-MAVS pathway, P1’s SV40-fibroblasts clearly had low levels of *IFNB*, *IFNL1*, and *IFIT1* production, consistently below those of any of the other fibroblasts with *TBK1* mutations (G159A/WT or W619*/W619*, from two previously reported patients—one with HSE and the other with systemic autoinflammation (8, 14)—tested here; Fig. 3 B), although the difference between P1’s and other TBK1-mutated SV40-fibroblasts did not reach statistical significance in these experiments. *IFNB* levels remain low in P’s SV40-fibroblasts, at ∼1/5 of its levels in healthy controls’ cells, at baseline or following HSV-1 infection at a multiplicity of infection (MOI) of 1 for 8 h (Fig. 3 C), whereas *IFNB* levels became about twice higher in P1’s cells than that of the healthy controls likely due to the enhanced HSV-1 replication levels in P1’s cells at this time point. Indeed, P1’s fibroblasts exhibited enhanced HSV-1 replication after HSV-1 infection at a MOI of 0.001, at levels similar to other fibroblasts with *AD TBK1* deficiency (G159A/WT) or AR complete IFNAR1 deficiency from other patients with HSE but much higher than that in healthy controls’ cells (Fig. 3 D). Pretreatment with exogenous IFN-β conferred resistance to HSV-1 infection in both AD and AR TBK1-deficient patients’ fibroblasts but not in IFNAR1-deficient fibroblasts (Fig. 3 D). These findings raise the possibility that homozygosity for the R308* TBK1 mutant might underlie a more severe impairment of cell-intrinsic antiviral I-IFN immunity mediated by various pathways than observed for the previously reported TBK1 mutants (whether present in the heterozygous or homozygous state), potentially accounting for the more severe course of the recurrent viral infections in this patient. Future investigations are required to clarify this point.

## Discussion

Unlike the four previously reported patients with AR TBK1 deficiency and systemic autoinflammation, including early-onset arthritis and vasculitis (14), the patient with AR TBK1 deficiency described here had recurrent confirmed or suspected viral infections and no autoinflammation. The patient experienced recurrent viral infections of the lungs and, despite undergoing hematopoietic stem-cell transplantation, the clinical findings at the time of death were consistent with post-viral encephalitis. The viral infectious phenotype of this patient is reminiscent of that observed in patients with AD TBK1 deficiency (8, 13). In the fibroblasts of our patient, homozygosity for the R308* variant was associated with an abolition or severe impairment of the induction of I- and III-IFNs following TLR3 or RIG-I/MDA5-MAVS stimulation. The R308* variant is located in the kinase domain and would be expected to destroy the structure and function of the protein due to the introduction of a premature stop codon. The very severe impairment of the I-IFN–related function of TBK1 observed in our experimental studies may explain the susceptibility of this patient to recurrent severe viral infections.

I-IFNs define a broad first-line host defense mechanism against various viruses, including HSV-1. This function has been shown to be crucial through studies of patients with defects of I-IFN signaling, including those experiencing unusually severe HSV-1 infections, such as HSE (10, 17, 18). Genetic defects of the TLR3–IFN induction pathway, including AD TBK1 deficiency, have been identified in children with HSE (8). Intriguingly, all four previously reported patients with AR TBK1 deficiency had neurological symptoms, including mild intellectual disability and epileptic seizures, but MRI scans of the central nervous system (CNS) were available for only one of these patients and they disclosed multiple white matter lesions, enlarged ventricles, and brain matter atrophy (14). However, no relationship was established between the genetic variants present in these patients and their CNS diseases. Anti-TNF treatment did not improve the CNS symptoms of these patients, who may have suffered from viral encephalitis. In our patient, MRI of the CNS revealed signs suggestive of post-viral ADEM. Our patient died of sudden respiratory arrest, probably due to involvement of the pons. In cases of neurological involvement in patients with TBK1 deficiency, CNS imaging should be performed, and treatment should be adjusted accordingly.

In our patient, there were a few findings suggestive of an inflammatory cause of the fever attacks. These attacks resolved within 2 days, during which serum c-reactive protein (CRP) and amyloid A concentrations increased only slightly. The surprising discrepancy between the two clinical phenotypes in patients with AR TBK1 deficiency is difficult to explain but may reflect differences in the biological impact of the TBK1 variants in the two studies. Another patient homozygous for an essential splice site mutation of *TBK1* died of critical COVID-19 pneumonia with central nervous system complications (16), a situation not unlike that reported here, although the deleteriousness of the TBK1 variant implicated has not been tested experimentally. In any case, a diagnosis of AD or AR TBK1 deficiency should be considered in patients with severe viral infections with involvement of one or more organs.

## Materials and methods

### Human subjects

Informed consent was obtained in Turkey, in accordance with local regulations and protocol for research on human subjects approved by the Institutional Review Board (IRB) of the medical faculty of Bursa Uludag University. Experiments were conducted in the United States, in accordance with local regulations and with the approval of the IRB of The Rockefeller University. Approval was obtained from the Rockefeller University Institutional Review Board in New York, NY, USA.

### Cell culture

Primary human fibroblasts were obtained from skin biopsy specimens from controls and P1, and were cultured in DMEM (GIBCO BRL, Invitrogen) supplemented with 10% fetal calf serum (FCS) (GIBCO BRL, Invitrogen). Immortalized SV40-transformed fibroblast cell lines (SV40-fibroblasts) were created by using 4 mg of a plasmid containing T-antigen DNA to transfect about 5 million cells by electroporation. The cells were then placed in two fresh 75 cm^2^ flasks, each containing 12 ml DMEM (GIBCO BRL, Invitrogen) supplemented with 10% FCS (GIBCO BRL, Invitrogen). SV40-fibroblast clones appeared after about 15 days. These clones were cultured and passaged for experimental use. HEK293T cells (ATCC) were maintained in DMEM supplemented with 10% FCS. All cells were regularly checked to ensure they were negative for mycoplasma.

### Sanger sequencing of genomic DNA

Genomic DNA samples from P1 and his parents were used as a template for the amplification of a 600–800 bp region encompassing the mutation by PCR with site-specific oligonucleotides. PCR was performed with 2 × Flash PCR MasterMix(Dye) (CW3009M; CoWin Biosciences). The primers used were: forward, 5′-AGC​CGT​GAA​AAC​AAC​TAC​CAG​A-3′; and reverse, 5′-GGT​GAA​GCT​GAG​GCA​TCT​TTT​C-3′. PCR products were purified by ultracentrifugation through Sephadex G-50 Superfine resin (Amersham-Pharmacia-Biotech), and sequenced with the Big Dye Terminator Cycle Sequencing Kit on an ABI Prism 3700 apparatus (Applied Biosystems). SnapGene was used for sequence analysis.

### Viral serological study by VirScan

Plasma was collected from P1 at 2 years of age. Serological tests for a group of common viruses were performed with the VirScan assay to assess the levels of antiviral IgG antibodies against various viruses, as previously described (16, 17, 18, 19, 20, 21). The z-score represents phage immunoPrecipitation sequencing (PhIP-Seq) antibody enrichment relative to mock immunoprecipitation for each of the viruses. Only those highly confident hits (i.e., z-score >3 in both technical replicates) are considered as real positives and shown.

### Western blots

HEK293T cells (5 × 10^5^ cells/ml) were used to seed a 12-well plate (1 ml/well). The following day, cells were transfected by incubation with 1.6 μg empty vector, WT, or mutant TBK1 plasmids for 24 h or 48 h in the presence of Lipofectamine 2000 Transfection Reagent (11668019; Invitrogen). HEK293T or SV40-fibroblast cell pellets were harvested, washed with phosphate-buffered saline and lysed in NP-40 lysis buffer (280 mM NaCl, 50 mM Tris, pH 8, 0.2 mM EDTA, 2 mM EGTA, 10% glycerol, 0.5% NP-40) supplemented with 1 mM DTT, PhosSTOP (Roche) and Complete Protease Inhibitor Cocktail (Roche). The protein lysate was subjected to SDS–PAGE and the resulting bands were transferred onto polyvinylidene difluoride membranes (Millipore Sigma). The membranes were blocked by incubation with 5% skim milk and incubated with primary antibodies overnight at 4°C. Primary antibodies against the following proteins were used in this study: TBK1 (N-ter) (ab40676; Abcam), TBK1 (C-ter) (#3504; Cell Signaling Technology), p-TBK1 (Ser172) (#5483; Cell Signaling Technology), IRF3 (66670-1-Ig; Proteintech), p-IRF3 (Ser396) (#4947; Cell Signaling Technology), Flag (A8592; Sigma-Aldrich). The membranes were then washed with Phosphate buffered saline with tween (PBST) and incubated with the corresponding horseradish peroxidase (HRP)–conjugated secondary antibodies at room temperature for 1 h. Protein signals were detected with an Amersham Imager 600 (GE Life Sciences) following incubation with Pierce ECL Western Blotting Substrate (32106; Thermo Fisher Scientific). Membranes were stripped and reprobed with HRP-conjugated anti-GAPDH antibody (HRP-60004; Proteintech), to control for protein loading.

### Cell stimulation

We used the synthetic double-stranded RNA analog poly (I:C) as a nonspecific agonist of TLR3 and MDA5/RIG-I. SV40-F were used to seed 24-well plates at a density of 90,000 cells/well and were stimulated for 6 h under two sets of conditions: (1) incubation with 25 μg/ml poly (I:C) alone to activate TLR3 signaling, and (2) transfection with 25 μg/ml poly (I:C) in the presence of Lipofectamine 2000 (11668019; Invitrogen) to activate MDA5/RIG-I signaling. After 6 h, cells were harvested for RNA extraction and assessed by reverse transcription-quantitative PCR (RT-qPCR).

### RT-qPCR

Total RNA was isolated from HEK293T cells and SV40-fibroblasts with the Quick-RNA MicroPrep Kit and Zymo-Spin IC Columns (#R1051; Zymo Research), according to the manufacturer’s protocol. The RNA was reverse-transcribed with the Super Script IV VILO (SSIV VILO) Master Mix (11756050; Thermo Fisher Scientific) according to the manufacturer’s instructions. RT-qPCR was performed with Applied Biosystems TaqMan assays for TBK1 (HS00179410_m1), IFNB1 (Hs01077958_s1), IFNL1 (Hs00601677_g1), IFIT1 (Hs00356631_g1), IL6 (Hs00174131_m1), and the β-glucuronidase (GUS; #4310888E) housekeeping gene for normalization. Results are expressed according to the ΔΔCt method, as described by the manufacturer.

### Luciferase reporter assays

HEK293T cells (2.5 × 10^5^ cells/ml) were used to seed a 96-well plate (100 μl/well). The following day, cells were transfected in triplicate with WT and mutant TBK1 plasmids, along with 100 ng of ISRE, IRF3, or NF-κB promoter-firefly luciferase reporter plasmid per well. The cells were also cotransfected with *Renilla* luciferase reporter plasmids (10 ng for ISRE, 10 ng for IRF3, and 50 ng for NF-κB) as an internal control. Transfections were performed with Lipofectamine 2000 Transfection Reagent (11668019; Invitrogen), according to the manufacturer’s protocol. After 24 h, luciferase activities were measured with a Dual-Luciferase Reporter Assay System (E2940; Promega). Firefly luciferase signals were normalized against *Renilla* luciferase signals to account for transfection efficiency.

### Viral infections and quantification of viral replication

SV40-immortalized fibroblasts (SV40-F; 5 × 10^4^ cells/well) were seeded into 48-well plates and infected with WT HSV-1 (KOS strain, VR-1493; ATCC) at an MOI of 0.001. Infections were carried out in DMEM containing 2% FCS for fibroblasts. After a 2-h adsorption period, cells were washed and replenished with 250 μl of fresh medium. At designated time points after infection, both supernatants and cells were collected and stored at −80°C for subsequent analysis. Viral titers were determined by TCID_50_ (median tissue culture infectious dose) per milliliter, as previously described (8, 22).

### Statistical analysis

Data are expressed as either mean ± standard deviation (SD) or mean ± standard error of the mean (SEM), as specified in figure legends. Statistical comparisons between control and mutant groups were conducted using one-way ANOVA with Tukey’s post hoc test applied for multiple group comparisons. For experiments involving repeated measurements, linear mixed-effects modeling was employed on log-transformed data to account for intra-sample variability. All statistical tests were carried out using GraphPad Prism 9 (v9.1.1). Significance thresholds are indicated in figures as follows: ns (P > 0.05), *P < 0.05, **P < 0.01, ***P < 0.001, and ****P < 0.0001.

## Supplementary Material

SourceData F2 is the source file for Fig. 2.

SourceData F3 is the source file for Fig. 3.

## Data Availability

All data are either displayed in the figures of the paper, or available as supplementary materials. Additional original raw data are available from the authors upon request.
