# Supplementary figures and images for: Recurrent severe viral infection in a child with inherited complete TBK1 deficiency
Source: J Hum Immun. 2025 Dec 3;2(1):e20250058. doi: 10.70962/jhi.20250058 (PMC12829739; doi:10.70962/jhi.20250058)

Fig.2B

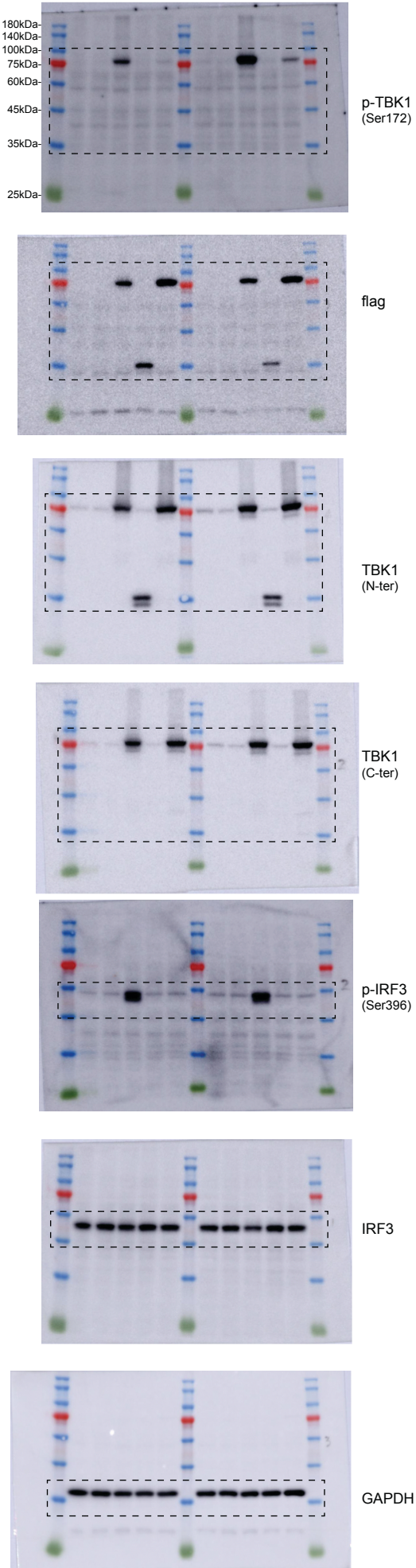

Fig.2C

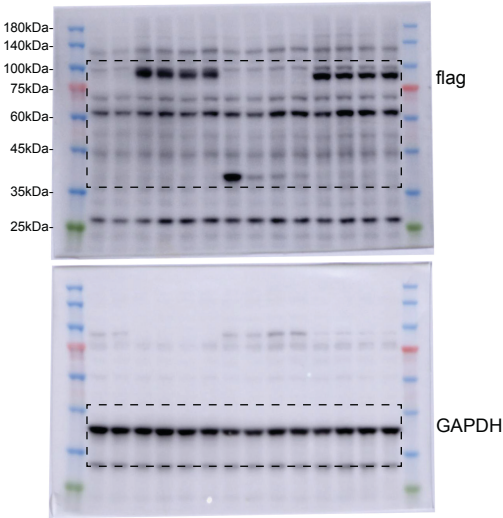

Supplement: SourceData F2 — is the source file for Fig. 2. [file jhi_20250058_sourcedataf2.pdf]

Fig.2B

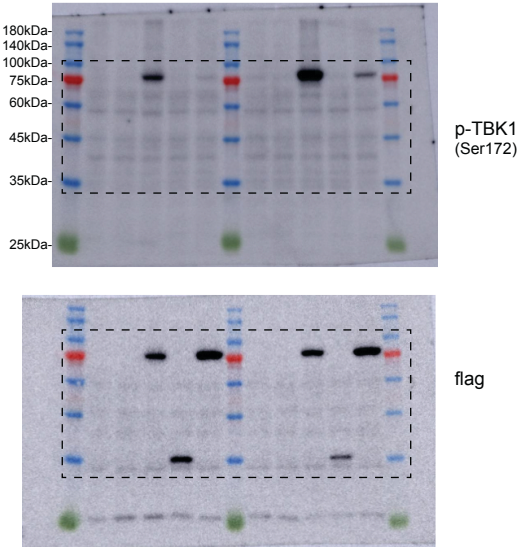

Fig.2C

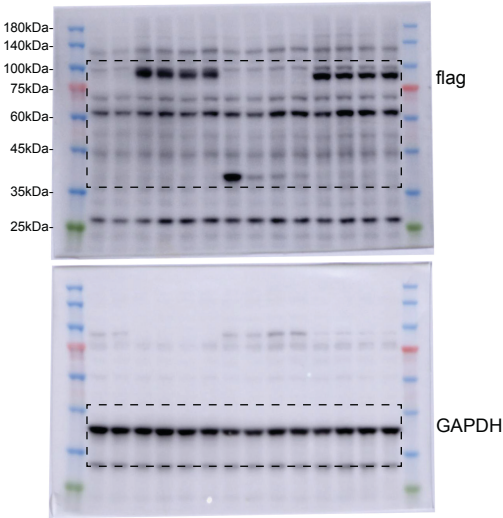

Fig.3A

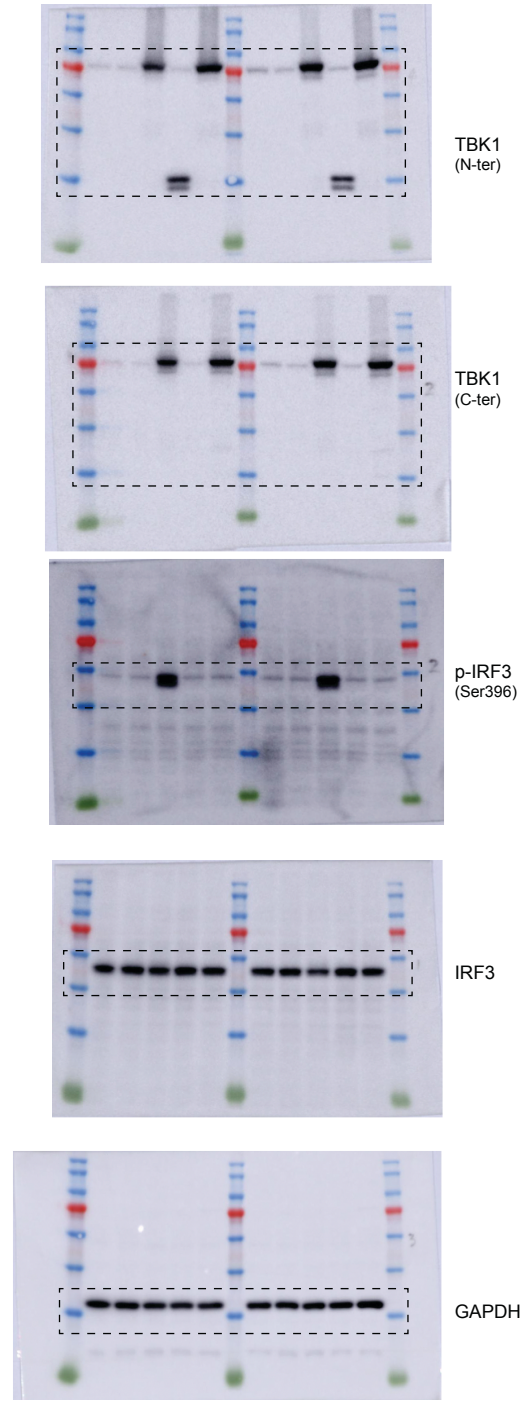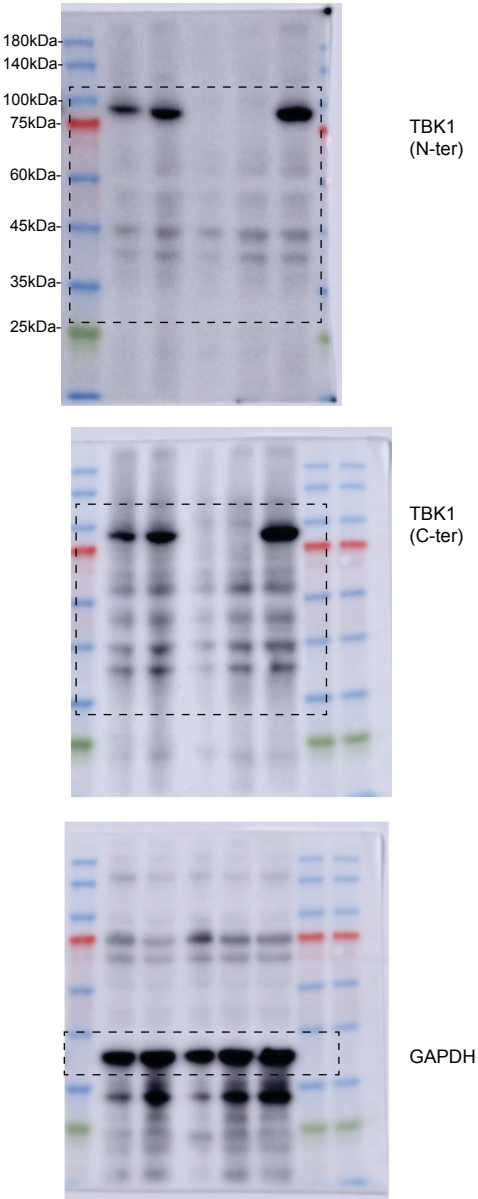

Supplement: SourceData F3 — is the source file for Fig. 3. [file jhi_20250058_sourcedataf3.pdf]
